# Cryocure-VT: the safety and effectiveness of ultra-low-temperature cryoablation of monomorphic ventricular tachycardia in patients with ischaemic and non-ischaemic cardiomyopathies

**DOI:** 10.1093/europace/euae076

**Published:** 2024-04-07

**Authors:** Atul Verma, Vidal Essebag, Petr Neuzil, Katia Dyrda, Jippe Balt, Borislav Dinov, Angeliki Darma, Arash Arya, Frederic Sacher, Vivek Y Reddy, Lucas Boersma, Ilya Grigorov, Tom De Potter

**Affiliations:** Division of Cardiology, McGill University Health Centre, D13.173, 1650 Cedar Avenue, Montreal, QC H3G 1A4, Canada; Division of Cardiology, McGill University Health Centre, D13.173, 1650 Cedar Avenue, Montreal, QC H3G 1A4, Canada; Department of Cardiology, Na Homolce Hospital, Prague, Czech Republic; Department of Medicine, Montreal Heart Institute, Montreal, QC, Canada; Department of Cardiology, St. Antonius Hospital, Nieuwegein, The Netherlands; Department of Electrophysiology, Leipzig Heart Center, Leipzig, Germany; Department of Electrophysiology, Leipzig Heart Center, Leipzig, Germany; Department for Internal Medicine, University Hospital Halle, Halle, Germany; Bordeaux University Hospital, IHU LIRYC, University of Bordeaux, Bordeaux, France; Department of Cardiology, Na Homolce Hospital, Prague, Czech Republic; Helmsley Electrophysiology Center, Mount Sinai Fuster Heart Hospital, New York, NY, USA; Department of Cardiology, St. Antonius Hospital, Nieuwegein, The Netherlands; Department of Heart Failure and Arrhythmias,Amsterdam University Medical Center, Amsterdam, The Netherlands; Adagio Medical, Inc., Laguna Hills, CA, USA; Cardiovascular Center, OLV Hospital, Moorselbaan 164, 9300 Aalst, Belgium

**Keywords:** Ventricular tachycardia, Ablation, Cryoablation, Ultra-low, Clinical trial, Outcomes

## Abstract

**Aims:**

The ultra-low-temperature cryoablation (ULTC) ablation system using −196°C N_2_ cryogen has been reported to create lesions with freeze duration–dependent depth titratable to over 10 mm with minimum attenuation by scar. Cryocure-VT (NCT04893317) was a first-in-human clinical trial evaluating the safety and efficacy of a novel, purpose-built ULTC catheter in endocardial ablation of scar-dependent ventricular tachycardias (VTs).

**Methods and results:**

This prospective, multi-centre study enrolled patients referred for *de novo* or second ablations of recurrent monomorphic VT of both ischaemic and non-ischaemic aetiologies. Primary safety and efficacy endpoints of the study were freedom from device- or procedure-related major adverse events (MAEs) up to 30 days post-ablation, acute non-inducibility of clinical VTs at the end of the procedure, and freedom from sustained VT or implantable defibrillator intervention at 6 months. Ultra-low-temperature cryoablation was performed in 64 patients (age 67 ± 11 years, 78% ischaemic, ejection fraction = 35 ± 10%) at 9 centres. The primary acute effectiveness endpoint was achieved in 94% (51/54) of patients in whom post-ablation induction was attempted. There were no protocol-defined MAEs; four procedure-related serious adverse events resolved without clinical sequelae. At 6-month follow-up, 38 patients (60.3%) remained VT-free, and freedom from defibrillator shock was 81.0%, with no significant difference between ischaemic and non-ischaemic cohorts. In 47 patients with defibrillator for at least 6 months prior to the ablation, the VT burden was reduced from median of 4, inter-quartile range (IQR, 1–9) to 0, IQR (0–2).

**Conclusion:**

In this first-in-human multi-centre experience, endocardial ULTC ablation of monomorphic VT appears safe and effective in patients with both ischaemic-cardiomyopathy and non-ischaemic-cardiomyopathy.

**Clinical Trial Registration:**

NCT04893317.

## Graphical Abstract

**Graphical Abstract:**
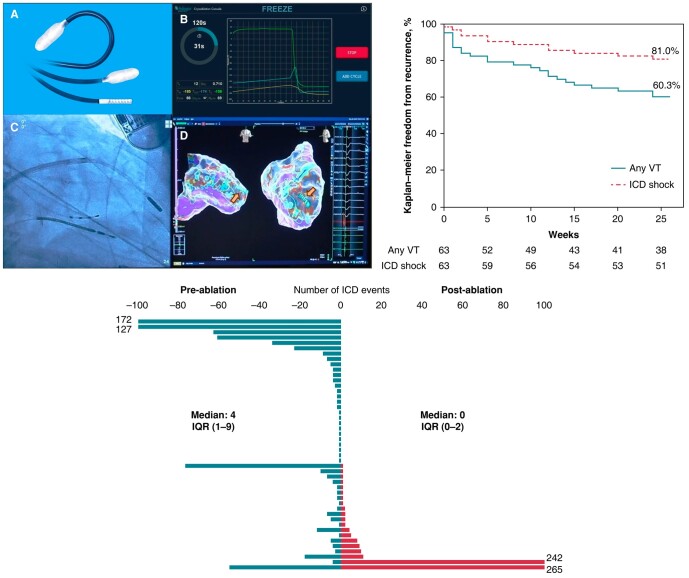

**What’s new?**

- Ultra-low-temperature cryoablation (ULTC) may provide increased tissue depth and scar penetration to improve outcomes for ventricular tachycardia (VT) ablation.
- Cryocure-VT was a single-arm, first-in-human study evaluating a new ULTC catheter designed specifically for VT ablation in ischaemic and non-ischaemic cardiomyopathy (*n* = 64).
- The trial demonstrated that the clinical VT was non-inducible in 94% of the patients at the end of the procedure.
- At 6-month follow-up, 60.3% remained VT-free, and freedom from ICD shock was 81.0%, with no significant difference between ischaemic and non-ischaemic cohorts.
- There were four serious adverse events.

## Introduction

The number of ventricular tachycardia (VT) ablation procedures has been increasing steadily over the last decade,^1–3^ driven in part by the technological advances in high-density mapping^1,2^ but counterbalanced by the persistent rate of acute complications.^1^ Furthermore, current irrigated-tip radiofrequency (RF) ablation catheters are not an ideal tool for performing VT ablation. Depths of RF lesions can be attenuated down to 3–5 mm by the presence of the scar,^1–3^ whereas wall thickness in most areas of the ventricle ranges from 6 to 12 mm. This limits the effectiveness of RF ablations beyond immediate endocardial and sub-endocardial layers, necessitating adjunctive use of epicardial ablations as well as development of other technically complex multi-catheter approaches.^2–4^

Ultra-low-temperature cryoablation (ULTC) may offer potential advantages for VT ablation over conventional RF techniques. Ultra-low-temperature cryoablation uses ‘near-critical’ nitrogen refrigerant near its boiling temperature of −196°C.^5^ Such ‘near-critical’ nitrogen combines the flow properties of gas with the density and thermal capacity of liquid, enabling uninterrupted injection through small lumen catheters. In pre-clinical models, ULTC produces lesions that can be titrated from 4 to over 10 mm in depth and can penetrate chronic scar.^6^ This would make it an ideal candidate for ablation of VT. However, there is a lack of human data to support this hypothesis.

The purpose of this study is to assess both the acute and long-term outcomes of VT ablation in a first-in-human, multi-centre trial of a novel ULTC system, comprised of the VT ablation catheter and the cryoablation console.

## Methods

### Trial design

Cryocure-VT (NCT04893317) is a prospective, single-arm, multi-centre clinical trial evaluating the safety and performance of a novel ULTC-based ablation system for treatment of VT. At each centre, local ethics review committees approved the study, which was performed in accordance with the Declaration of Helsinki. Primary safety and effectiveness endpoint events were blindly adjudicated. An independent Data and Safety Monitoring Board (DSMB) was utilized. The original protocol was conceived and designed, written by the sponsor, and then refined with input from the principal investigators. Data analysis was performed by the sponsor, and data interpretation was provided by the principal investigators (A.V. and T.D.P.). All principal investigators members approved the data analyses and interpretation, manuscript contents, and the decision to publish.

### Study participants

Patients were enrolled in nine centres in seven countries (see Supplementary material online, *Appendix*). Inclusion criteria were age ≥18 years; indication for catheter ablation of drug-refractory, symptomatic monomorphic VT; ischaemic cardiomyopathy (ICM) or non-ischaemic (NICM) cardiomyopathy; first or second ablation procedure; and presence of implantable cardioverter defibrillator (ICD) prior to or immediately after the ablation. Major exclusion criteria included ejection fraction <20%; New York Heart Association Class IV heart failure (HF); recent (<60 days) acute coronary syndrome, cardiac surgery, or percutaneous coronary intervention; intra-cardiac thrombus; or prior ablation within 4 weeks. Detailed inclusion and exclusion criteria are provided in the Supplementary material online, *Appendix*. All patients provided written informed consent prior to study procedures.

### Ablation procedure

All procedures were performed under deep sedation or general anaesthesia as per local institutional practice. Venous and/or arterial access was obtained via femoral puncture. Access to the left ventricle (LV) was obtained via the retrograde aortic or transseptal approach or both. Patients were immediately heparinized to maintain an activated clotting time of >300 s. All procedures were guided by a three-dimensional electroanatomical mapping system, and a multi-polar mapping catheter was used to perform baseline voltage mapping of the LV and/or right ventricle where applicable. Voltage criteria were used to define endocardial and epicardial scar^7^ based on both bipolar and unipolar electrograms.^8^ Investigators could use any mapping technique in conjunction with voltage mapping including (and not limited to) activation mapping during VT, pace mapping, identification of isochronal late mapping zones, and identification of late systolic potentials or local abnormal ventricular activity.

**Figure 1:**
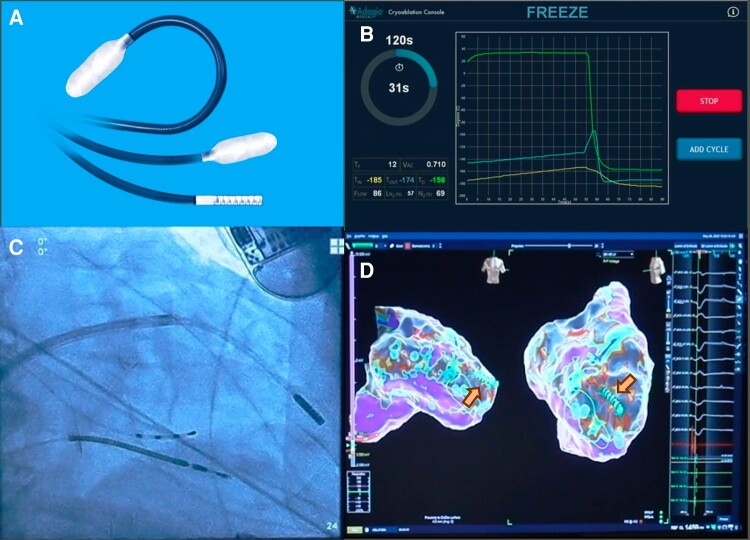
Ultra-low-temperature cryoablation system for VT ablations. (*A*) Catheter ablation element, showing deflection and ice ball formation; (*B*) screen of the cryoablation console showing temperature profile during typical cryoablation energy application; catheter image on fluoroscopy (*C*) and electroanatomic map (*D*). VT, ventricular tachycardia.

**Figure 2:**
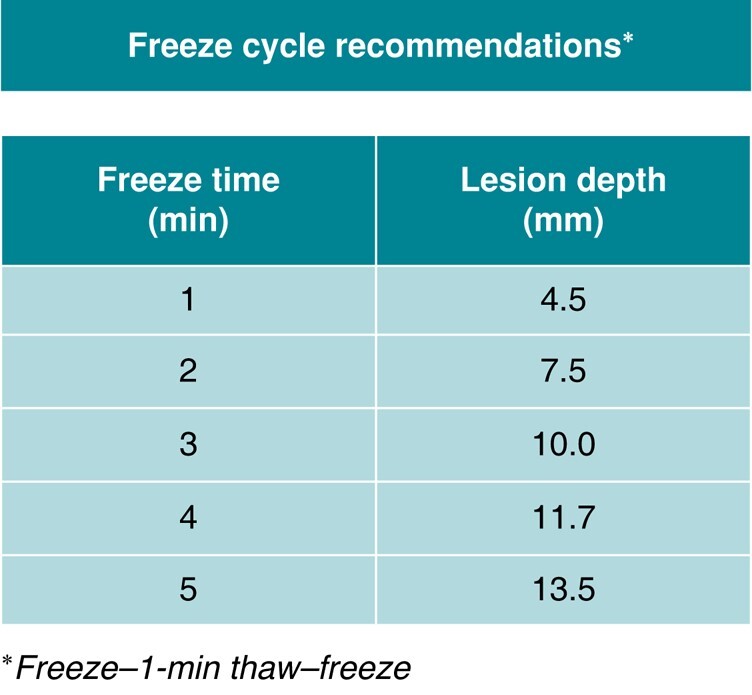
Freeze cycle recommendation utilized in the study. The following freeze times are based on a freeze–thaw–freeze cycle where each freeze is the time specified below. The table below is reproduced from De Potter *et al*,^10^ copyright authors. Reprinted with permission.

Ablation was performed using the ULTC ablation system (Adagio Medical Inc., Laguna Hills, CA, USA) consisting of the commercially available ULTC console capable of supporting both atrial^9^ and ventricular ablations and a purpose-built VT ablation catheter. The catheter (vCLAS™, Adagio Medical Inc.) features a 9 Fr, bi-directionally deflectable shaft (50 mm diameter, >180° deflection) and a solid 15 mm long cryoablation element with eight 1 mm electrodes for pacing and intracardiac electrograms (IEGM) recording (*Figure 1*). The VT ablation catheter is compatible with any 10 Fr fixed-curve or steerable introducer sheath for both transseptal and retrograde access to the LV and can be integrated with commercial, impedance-capable electroanatomic mapping systems. The depths and lateral dimensions of the lesions are dependent on the duration of a freeze–thaw–freeze cycle (see Supplementary material online, *Appendix*) where the freeze durations range from 1 to 5 min and the thaw is 1 min (*Figure 2*). The presence of ischaemic scar does not appear to significantly affect the depth of ULTC lesions in animal models.^6,11^ Investigators estimated wall thicknesses at ablation target zones using pre-procedural computed tomography (CT) or magnetic resonance imaging or intra-procedural intra-cardiac echocardiography (ICE) where available. Importantly, to maximize the energy transfer and achieve target lesion depth, the entire length of cryoablation element was positioned in contact of the tissue, as ascertained by surrogates for tissue contact such as fluoroscopic appearance of catheter bending, the catheter shadow on electroanatomic map, ICE imaging, and catheter IEGMs. Regions targeted for ablation were left to the discretion of the operator but could include an identified critical isthmus for a VT or a substrate-based approach.

Pre-ablation and post-ablation VT inductions by programmed ventricular pacing were performed in accordance with each institution’s standard of care, usually with two-drive cycle length and a minimum of three extra stimuli down to refractory period. ICD programming was left to the discretion of the investigators, with the general recommendation to follow recent Heart Rhythm Society (HRS) guidelines.^12^

### Follow-up

Prior to discharge, patients were evaluated for any early post-procedural complications. Follow-up visits occurred at 30 days, 3, and 6 months. Device interrogation was performed at each follow-up visit for arrhythmia documentation. Continuation of anti-arrhythmic medications was left to investigator discretion, but the protocol did not mandate any medication discontinuation. Oral anticoagulation for the first 1–2 months post-ablation was encouraged.

### Endpoints

The primary safety endpoint was freedom from any major adverse events (MAEs) within 30 days of the procedure as defined in the Supplementary material online, *Appendix*. All adverse events were adjudicated for relatedness to the device and procedures by the independent DSMB (Supplementary material online, *Appendix*). The primary acute efficacy endpoint was the proportion of patients with non-inducible clinical VT at the conclusion of the ablation procedure. The primary clinical effectiveness endpoint was 6-month freedom from sustained VT (>30 s) or appropriate ICD intervention (shock or anti-tachycardia pacing). Secondary endpoints included the proportion of patients non-inducible for any monomorphic VT at the end of the procedure, reduction of VT burden at 6 months, and freedom from device- or procedure-related serious adverse events (SAEs).

### Statistical analysis

Given that this was a first-in-human trial, no formal sample size calculation was performed. A sample of more than 60 patients is consistent with that of in many VT ablation trials. Continuous variables were reported as mean ± standard deviation or median (25th and 75th percentile, minimum and maximum) and were compared by a student *t*-test or Mann–Whitney U test. Categorical variables were reported as counts (percentage) and were compared using Pearson χ^2^ test, Fisher’s exact test, and logistic regression. The primary effectiveness outcome was evaluated with Kaplan–Meier analysis and the log-rank test. The presence and clinical nature of inducible VTs at the end of the procedure were assessed by the treating physician. Ventricular tachycardia burden assessment was performed by counting all sustained VT episodes (>30 s) inclusive of monitoring zone VTs and all ICD therapy deliveries (shock and anti-tachycardia pacing [ATP]). For episodes of VT storm (>2 h of continuous VT of the same cycle length within a 24-h period), we counted every delivered shock as a therapy as well as any ATP that broke the VT. Clusters of multiple ATPs that did not break the VT, occurring within 30 min of each other, were counted as a single ATP event. A *P*-value <0.05 was considered statistically significant. All analyses were conducted with Minitab Statistical Software (Minitab LLC, State College, PA, USA).

## Results

### Patient characteristics

A total of 64 patients underwent ablation. Baseline characteristics are shown in *Table 1*. Most patients had underlying ischaemic cardiomyopathy (78.1%) with the rest having non-ischaemic cardiomyopathy. For all but two patients, the study procedure was their first VT ablation. One patient withdrew consent shortly after the procedure and was excluded from the chronic effectiveness analysis. Of the remaining 63 patients, 47 (75%) had an ICD implanted for longer than 6 months prior to the ablation; an additional 9 patients received their ICDs 75–143 days before the procedure; and the rest were implanted post-ablation.

**Table 1:** Epidemiology

	*n* = 64^a^
Age, years	67 ± 11
Male sex	95.3% (61/64)
LVEF	35 ± 10%
≤30%	27 (42.2%)
31–50%	35 (54.7%)
>50%	2 (3.1%)
CAD	81.3% (52/64)
Prior MI	78.1% (50/64)
Diabetes mellitus	29.7% (19/64)
Hypertrophic cardiomyopathy	6.3% (4/64)
Congestive heart failure	57.8% (37/64)
Valvular disease	18.8% (12/64)
Hypertension	82.8% (53/64)
Medications prior to ablation	
Beta-blockers	52 (81.3%)
Class I anti-arrhythmics	4 (6.3%)
Amiodarone	27 (42.2%)
Solatol	13 (20.3%)

LVEF, left ventricular ejection fraction; MI, myocardial infarction; CAD, coronary artery disease.^a^ One patient withdrew consent and is excluded from chronic effectiveness analysis.

### Procedural details

Procedural strategies for 64 patients are shown in *Table 2*. Deep sedation was used in 28 (43.7%) and general anaesthesia in 36 (56.3%) patients. All ablations were performed under electroanatomical guidance using Biosense-Webster Carto system in 24 (37.5%) and Abbott Ensite NavX system in 40 (62.5%) of the cases, in accordance with institutional VT ablation practices. Intracardiac echocardiography was used in 44 (68.8%) procedures to image potential thrombus, to assist with catheter positioning, and to confirm the depth of the targeted tissue. Substrate-based mapping and substrate-based ablation were by far the most common approaches utilized in 84 and 87% of the patients, respectively.

**Table 2:** Anaesthesia, mapping, and ablation strategies, *n* = 64

Anaesthesia (*n*, %)	
General anaesthesia	36 (56.3%)
Deep sedation	28 (43.7%)
Mapping strategies (*n*, %)	
Substrate mapping	54 (84.4%)
Pace mapping	27 (42.2%)
Activation mapping	20 (31.3%)
Entrainment mapping	4 (6.3%)
ICE/Cartosound	2 (3.1%)
Ablation strategies and targets (*n*, %)	
Substrate-based ablation/homogenization	56 (87.5%)
VT exit site	25 (39.1%)
Scar de-channelling	12 (18.8%)
Slow conduction channel	11 (17.2%)
VT isthmus	8 (12.5%)
LAVA elimination	7 (11.0%)

ICE, intracardiac echocardiography; VT, ventricular tachycardia; LAVA, local abnormal ventricular activity

**Figure 3:**
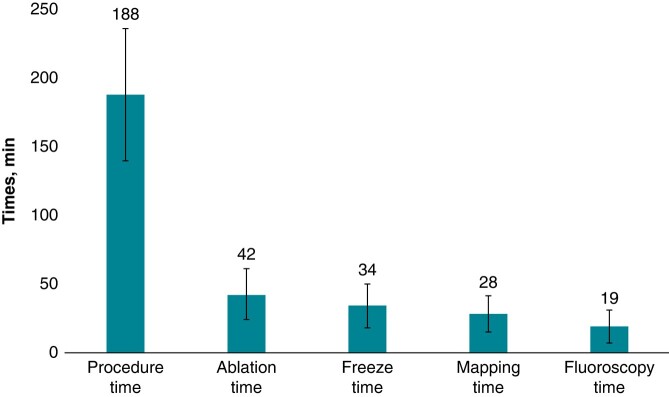
Procedure times. The data closely match prior reports for initial multi-centre experience cohorts (*n* = 13).^10^

An average of 8.9 ± 4.3 [median 8, inter-quartile range (IQR) (6–10)] ULTC ablations were performed per patient with 62% of 570 total lesions utilizing the 2-min protocol, 19% utilizing the 3-min protocol, and 13% utilizing the 1-min protocol. Only 10 lesions in 4 patients exceeded 3-min duration, and only 2 patients had lesions with <1-min duration. The average freeze duration (inclusive of two cryo applications in the freeze–thaw–freeze protocol) per lesion was 3.8 ± 1.2 min. Total procedure and associated times are shown in *Figure 3*.

### Safety analysis

The primary safety event (defined MAEs in Supplementary material online, *Appendix*) occurred in 0% of the patients. Four SAEs are shown in *Table 3*. An asymptomatic ventricular pseudo-aneurysm over the ablation site with 2 mm wall thickness in one patient was an incidental finding in a post-procedural CT and resolved without clinical sequelae. There were two small, haemodynamically irrelevant pericardial effusions, one of which was drained post-procedurally. During the drain insertion, there was an accidental arterial injury, resulting in an extended hospital stay but the patient recovered. One patient experienced haemodynamic instability requiring extracoporeal membrane oxygenation (ECMO) support during the procedure due to incessant VTs induced during initial mapping (nine different morphologies) that were successfully eliminated by ablation. One patient expired on Day 182 post-ablation due to decompensated HF.

**Table 3:** Adjudication of device- and procedure-related serious adverse events

Event type	No. of events	Percentage
Asymptomatic false aneurysm in 2 mm tissue	1	1.6
Small pericardial effusion	2	3.1
Haemodynamic instability during procedure	1	1.6
Total	4	6.3

### Acute effectiveness analysis

The results of primary acute effectiveness analysis are summarized in *Table 4*. An average of 2.1 ± 1.3 VTs were induced in 61 patients with successful pre-procedural induction, of which 47% were haemodynamically stable and 83% were deemed to be the clinical target VT. Eighteen (28%) patients had haemodynamically stable VTs only, 31 (48%) had haemodynamically unstable VTs only, and 9 (14%) patients had a mix of stable and unstable morphologies, with stability data missing in 3 patients (not inducible at start). The average cycle length of the induced clinical VTs was 371 ± 187 ms. Post-ablation re-induction was attempted in 54 patients (88%). The re-induction was not performed due to failure of the initial induction in three patients (two ICM) or concerns about haemodynamic stability in seven patients (all ICM).

**Table 4:** Primary acute effectiveness analysis

Acute inducibility data	
Patients with pre-procedural inducibility	95.3% (61/64)
# VTs induced/patient	2.1 ± 1.3
% Haemodynamically stable VTs	46.8% (59/128)
% Clinical VTs	82.9% (111/134)
Cycle length of clinical VTs	371 ± 187 ms
Patients with attempted post-procedural reinduction	84.4% (54/64)
Freedom from clinical VT	94.4% (51/54)
Freedom from any VT	85.2% (46/54)
Eliminated clinical VTs	97.1% (100/103)

VT, ventricular tachycardia.

The primary acute effectiveness endpoint of freedom from re-induced clinical VT was reached in 94.4% (51/54) of patients with a total of 97.1% (100/103) clinical VTs eliminated. The secondary acute effectiveness endpoint of freedom from any re-induced VT was reached in 85.2% (46/54) of patients. Among the site-specific procedural variables, use of ICE was associated with acute success (*P* = 0.035), as all re-inductions of clinical VTs occurred in cases without the documented use of ICE.

### Six-month effectiveness analysis

**Figure 4:**
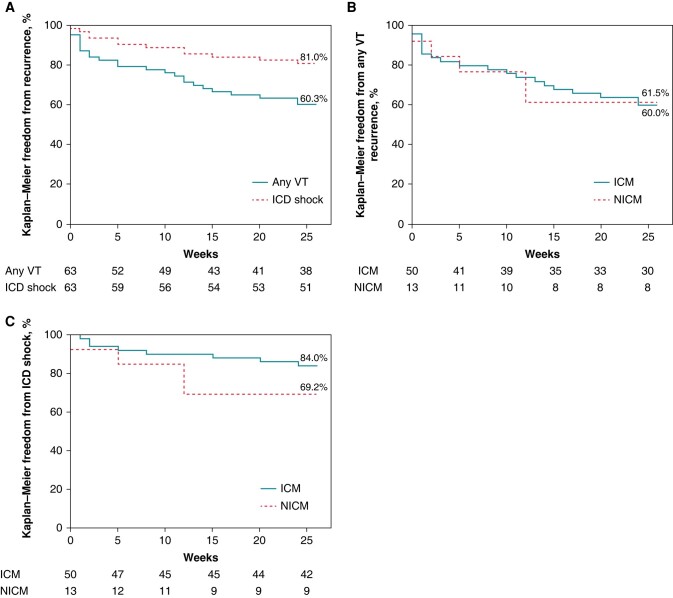
Kaplan–Meier freedom from sustained VT or appropriate ICD intervention. (*A*) Comparison of freedom from any recurrence and freedom from ICD shock. (*B*) Comparison of freedom from any recurrence in patients with ICM and NICM. (*C*) Comparison of freedom from ICD shock in patients with ICM and NICM. ICD, implantable cardioverter defibrillator; VT, ventricular tachycardia.

Kaplan–Meier freedom from VT recurrence is shown in *Figure 4*. Twenty-five patients experienced the primary effectiveness endpoint at an average of 8 weeks post-ablation. The freedom from any VT recurrence was 60.3% (38/63) for the entire cohort, with no significant difference between patients with ischaemic and non-ischaemic cardiomyopathy (59 vs. 62%, respectively) or between the study sites. Of the 25 patients with recurrences, 8 (32%) had a single episode of VT or ICD therapy through the entire follow-up period.

Early (<30 days) recurrence occurred in 11 (17.5%) patients, of which 1 required readmission; 6 were treated by ATP alone; and 5 had ICD shock. Four of the 11 early recurrence patients had no further sustained VT episodes or ICD interventions through 6-month follow-up.

Overall freedom from ICD shock through 6-month follow-up was 81.0%, with no significant difference between ischaemic (84.0%) and non-ischaemic (69.2%) cardiomyopathy patients. Four patients had repeat ablations using non-study devices at an average of 84 days after the index procedure.

Among 63 patients followed chronically, amiodarone was present at baseline in 26 (*Table 1*) and was initiated immediately following the procedure or following recurrences in 7 and 2 patients, respectively. Among seven patients with amiodarone immediately post-procedure, all VTs were eliminated in 3, all clinical VTs in 1, and no re-induction was performed in 3. During follow-up, 14 (22%) patients remained on the original dose of amiodarone, while amiodarone was stopped or reduced in 13 (20.6%) and 8 (12.7%) patients, respectively, amounting to 60.0% (21/35) reduction in the overall use of amiodarone. The use of amiodarone declined in 61.5% (16/26) of patients with amiodarone at baseline and 55.6% (5/9) of patients who started amiodarone post-procedurally.

### Ventricular tachycardia burden analysis

**Figure 5:**
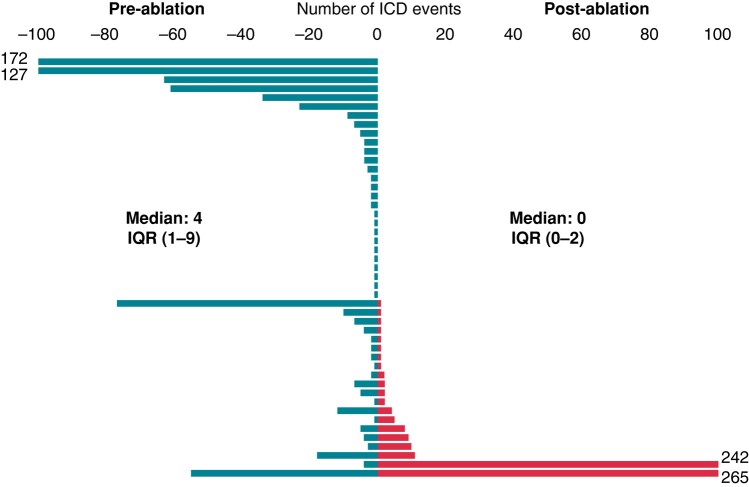
Ventricular tachycardia burden 6 months before and after ablation.

The frequency distribution of the 1320 arrhythmic events in 47 patients with ICD implanted for more than 6 months prior to the procedure is shown in *Figure 5*. The median number of events over the 6-month periods pre- and post-ablation was reduced from 4, IQR (1–9) to 0, IQR (0–2) [difference 2, 95% confidence interval (1–4)], with similar change in burden in patients with both ischaemic and non-ischaemic cardiomyopathy. Of two patients with substantial increase in burden (200+ events), one (NICM) presented with VT storm treated by ATP and shocks ∼12 weeks post-procedure and was re-ablated; the other patient (ICM) showed increasing burden of ATP-treated VTs starting Week 11 post-ablation and was re-ablated outside of 6-month follow-up window. Neither of these patients received amiodarone at any time in the study.

## Discussion

This is a first-in-human study describing the acute and 6-month outcomes for ULTC of VTs in patients with structural heart disease. We showed >90% acute elimination of clinical VT and 85% elimination of all inducible VTs. At 6 months, 60% of patients had no VT recurrence. The median VT burden over 6 months was reduced from four events pre-ablation to zero post-ablation. The freedom from ICD shock was 81%. There were no direct catheter-related MAEs but four SAEs (6%), which is in keeping with complication rates reported for prior VT ablation studies.^12^ Only one patient died in follow-up, due to HF.

The paucity of similar, single-device evaluation datasets, the inherent heterogeneity of VT patients enrolled in studies, and the lack of common reporting standards all make meaningful comparisons difficult. In the original Thermocool-irrigated RF catheter trial for treatment of ischaemic post-myocardial infarction (MI) patients, Stevenson *et al.*^14^ reported 49% acute success in eliminating all targeted VTs and 53% freedom from VT recurrence at 6 months. A subsequent post-market study in patients with coronary artery disease (CAD)^15^ demonstrated acute and chronic endpoints of 75% and 62%, respectively. In both studies, close to one-third of the patients had prior ablations, although that was not predictive of success.^16^ More recently, the LESS-VT trial (NCT03490201) of another irrigated RF catheter reported 93% acute success and 58% success at 6 months after a combination of endocardial and epicardial ablations in patients with non-ischaemic cardiomyopathy.^17^ Even in trials of very early VT ablation in patients with a first shock^18,19^ or preventative ablation ahead of receiving an ICD,^20^ freedom from VT ranges from 60 to 70%, and these may represent populations earlier in the disease than our population. In a somewhat more direct comparison with a novel deep myocardial lesion technology, a three-centre Canadian trial of a saline-enhanced RF needle catheter reported 92% acute success and 42% freedom from VT at 6 months in 25 patients with both ischaemic and non-ischaemic cardiomyopathy.^21^ The US trial of the same technology reported 73% acute and 48% chronic success at 6 months, albeit in cohort with much more advanced disease.^22^ In the context of these studies, our 94% rate of acute clinical VT success and the 85% rate of non-inducibility for all VT are on the higher end of prior reports. The 60% freedom from any VT and 81% freedom from ICD shock are also comparable or better than other studies despite this being a first-in-human experience with a new technology.

Current guidelines^21,23^ continue to view VT ablations as a second-line approach in patients with structural heart disease. This stems from the fact that both initial^24,25^ and more recent^18–20^ randomized trials of early VT ablation showed benefits in VT-specific endpoints but failed to demonstrate consistent impact on hard clinical endpoints like death and hospitalization. There is also a persistent 9–11% rate of major procedural complications,^12^ including death, stroke, cardiac perforations, tamponade, and acute HF decompensation that need to be balanced against the benefits of the reduced VT recurrence. In addition, 14–19% of patients are re-hospitalized within 30 days of VT ablation,^3,26,27^ with recurrent VT and HF representing many of those readmissions. In the US first-in-human trial of a saline-enhanced irrigated needle, 19% had SAEs including two procedural deaths and a 22% early re-hospitalization rate.^22^ In the current study, the 6% complication rate is quite consistent with other VT ablation studies. No patients died from procedural causes, only one patient (1.6%) had an early readmission for recurrent VT, and only one patient died in follow-up due to progressive HF. This is a reassuring result for a first-in-human trial.

There are other potential technological benefits of ULTC for VT ablation. One is the lack of catheter irrigation during the procedure. Fluid management is very important for this patient population with low EFs and a history of HF. Upwards of 1.5 L of irrigation fluid has been reported for RF ablations.^4,12,28^ This can be of particular concern when a substrate homogenization strategy is chosen, which is effective, but may double the ablation time vs. more targeted procedures.^21^ Procedural time was also on the lower end of other clinical experiences despite the novelty of the ablation system and may be due to the efficiency of the large lesion footprint. The consistency of the procedural characteristics reported here with those reported in the initial acute results^10^ highlights a short learning curve for all operators in the study. Ultra-low-temperature cryoablation lesions with 15+ mm length and 8–12 mm width^11^ may be particularly suitable for scar homogenization. The maximum number of 22 lesions used in this study was for homogenization of a very large basal to mid inferior septal to lateral scar after five VT morphologies were induced pre-ablation. Finally, the depth of ULTC lesions from the endocardial surface may benefit targeting of mid-myocardial substrates.

Trials offer mixed results on whether outcomes of ablations in patients with non-ischaemic cardiomyopathy are inferior to that of patients with ischaemic cardiomyopathy.^18,28,29^ Typically, the substrate in non-ischaemics is more mid-myocardial or epicardial making these cases more complex, requiring adjuvant epicardial access and/or bipolar ablation in 30–60% of cases.^13,23,30^ In this study, only endocardial ablation was performed in non-ischaemics with 100% acute elimination of the clinical VT and 83% acute elimination of all VTs, similar to the ischaemic patients. The 6-month freedom from VT recurrence and ICD shock were also similar between the groups. The small size of the non-ischaemic cohort, however, does not allow us to draw definitive conclusions, and larger studies, currently in planning, may help address these questions. Finally, when the catheter delivers cryothermy, it also adheres to the tissue, which increases catheter stability, but the impact of this on outcomes cannot be determined from this small study.

### Study limitations

This was a small, first-in-human study. Findings of this study will need to be further evaluated and confirmed in larger studies, which are planned in the USA, and once the catheter becomes commercially available. There was also no comparator arm using traditional RF ablation, and therefore, we cannot make direct comparisons between one technology and the other. The low number of female patients and relatively small cohort of NICM patients limit generalizability of the findings. Seven patients had amiodarone initiated immediately post-ablation, which could have affected the results, although freedom from antiarrhythmic use is not a typical primary endpoint for structural VT ablation trials. The inclusion criteria of this first-in-human study did not allow evaluation of the technology in patients presenting in electrical storm in whom the effective suppression of acute inducibility might be of particular benefit. Finally, the variation in institutional protocols for intra-procedural VT induction and ICD programming across nine enrolling centres, while representing a slice of the prevailing practices and not affecting performance endpoints in this study, may become significant in a larger patient population and should be addressed in future studies design.

## Conclusions

In this first-in-human multi-centre experience, endocardial ULTC ablation of monomorphic VT appears safe and effective in patients with both ICM and NICM.

## Supplementary Material

euae076_Supplementary_Data

## Data Availability

All relevant data are within the manuscript and its Supplementary material.
