# Supplementary material for: Cryocure-VT: the safety and effectiveness of ultra-low-temperature cryoablation of monomorphic ventricular tachycardia in patients with ischaemic and non-ischaemic cardiomyopathies
Source: Europace. 2024 Apr 7;26(4):euae076. doi: 10.1093/europace/euae076 (PMC10998960; doi:10.1093/europace/euae076)

Supplemental Material

Supplement to: Verma A, Essebag E, Neuzil P, et al, Cryocure-VT: The Safety and Effectiveness of Ultra-low Temperature Cryoablation of Monomorphic VT in Patients with Ischemic and Non-Ischemic Cardiomyopathies (CRYOCURE-VT trial).

I. Investigators and Centers (in order of recruitment)

McGill University Health Centre, Montreal, Quebec, Canada
Atul Verma, Vidal Essebag

Na Homolce Hospital, Prague, Czech Republic
Petr Neuzil, Vivek Reddy

Montreal Heart Institute, Montreal, Quebec, Canada
Katia Dyrda

St. Antonius Hospital, Nieuwegein, Netherlands
Jippe Balt, Lucas Boersma

Cardiovascular Center, OLV Hospital, Aalst, Belgium
Tom De Potter

Leipzig Heart Center, Leipzig, Germany
Borislav Dinov, Angeliki Darma

Southlake Regional Health Centre, Newmarket, Ontario, Canada
Atul Verma

University Hospital Halle, Halle, Germany
Arash Arya

Bordeaux University Hospital, IHU LYRIC, University of Bordeaux, Bordeaux, France
Frederic Sacher

II. Data and Safety Monitoring Board Members

David Donaldson

University of California, Irvine, California, USA

Reynolds Delgado

Baylor St. Luke’s Medical Center, Houston, Texas, USA

Tasneem Naqvi

Mayo Clinic, Phoenix, Arizona, USA

III. Detailed Inclusion and Exclusion Criteria

Inclusion Criteria:

- Male or female the ages of ≥ 18 years
- Eligible for a catheter ablation due to Ischemic and/or non-ischemic recurrent symptomatic sustained monomorphic Ventricular Tachycardia also defined as having a similar QRS configuration from beat to beat.
- Has or will be receiving an ICD prior to hospital discharge post procedure.
- Refractory to at least one AAD (Refractory is defined as an AAD not able to treat the arrhythmia satisfactorily or induces unwanted side effects).
- Subject has LVEF>20%, confirmed by echo or comparable technique in the previous 3 months or during baseline evaluation
- Willingness, ability, and commitment to participate in baseline and follow-up evaluations for the full length of the study
- Willingness and ability to give an informed consent

Exclusion Criteria:

- Any known objective contraindication to ventricular tachycardia ablation, TEE, or anticoagulation, including but not limited to the identification of any cardiac thrombus or evidence of sepsis
- Any duration of continuous arrythmia that is not monomorphic ventricular tachycardia. Multiple monomorphic tachycardia is acceptable, but polymorphic VT is not.
- Any VT ablation within 4 weeks prior to enrollment
- More than one prior (>4 weeks) Ventricular Tachycardia ablation or prior surgical treatment for ventricular tachycardia
- Ventricular tachycardia secondary to electrolyte imbalance, active thyroid disease, or any other reversible or non-cardiac cause
- Cardiovascular conditions, as described below:
  a. Class IV heart failure
  b. Aortic aneurysm
  c. Previous cardiac surgery or percutaneous coronary intervention within 60 days prior to the procedure
  d. Interatrial baffle, closure device, patch, or PFO occlusion device
  e. IVC filter
  f. Coronary artery bypass graft (CABG) procedure within six (6) months prior to the ablation procedure
  g. Severe Mitral or Aortic insufficiency or stenosis based on most recent TTE
  h. Cardiac myxoma
  i. Significant congenital anomaly
  j. Recent Myocardial Infarct (MI) or unstable angina, within 60 days prior to the ablation procedure
  k. Mechanical aortic or mitral valve
- Any previous history of cryoglobulinemia
- History of blood clotting or bleeding disease

- Any prior history of documented cerebral vascular accident (CVA), TIA or systemic embolism (excluding a post-operative Deep Vein Thrombosis, DVT), within 6 months prior to the ablation procedure.
- Breastfeeding, pregnant, or anticipated pregnancy during study follow-up
- Current enrollment in any other study protocol where testing or results from that study may interfere with the procedure or outcome measurements for this study
- Any other condition that, in the judgment of the investigator, makes the patient a poor candidate for this procedure, the study or compliance with the protocol (includes vulnerable patient population, mental illness, addictive disease, candidate for heart transplantation, patient with ventricular assist device, or terminal illness with a life expectancy less than 12 months)

IV. Study Endpoints

Primary Endpoints

- **Safety:** Proportion of subjects who are free from definite or probable device/procedure related Major Adverse Events (MAEs) that occur during or within 30 days following the cryoablation procedure.
- **Clinical Performance:** Proportion of subjects receiving a single cryoablation procedure with freedom from ventricular tachycardia lasting longer than 30 seconds or appropriate ICD intervention until the end of the 6 month follow up period.
- **Procedural Performance:** Proportion of subjects with non-inducible clinical monomorphic VT at the conclusion of the initial cryotherapy ablation procedure.

Secondary Endpoints

- The proportion of study subjects with probable or definite device or procedure related serious adverse events (SAEs), including MAEs as described above, or serious adverse device effects (SADEs) between 30 days up to 12 months post-procedure.
- The proportion of study subjects with non-inducible sustained monomorphic VT at the end of the ablation procedure.
- Reduction of VT burden at 6 months and 12 months.
- The proportion of study subjects with freedom from Ventricular Tachycardia lasting longer than 30 seconds or appropriate ICD intervention at 12 months without the use of anti-arrhythmic drugs (AADs)
- The proportion of study subjects with freedom from Ventricular Tachycardia lasting longer than 30 seconds or appropriate ICD intervention at 12 months with previously failed AADs.

V. Major Adverse Events Definitions In the Study

Major Adverse Event	Study Definition
Death	
Myocardial Infarction	Presence of any one of the following criteria:<br>• detection of ECG changes indicative of new ischemia (new ST-T wave changes or new LBBB) that persist for more than 1 hour;<br>• development of new pathological Q waves on an ECG<br>• imaging evidence of new loss of viable myocardium or new regional wall motion abnormality.
Cardiac Perforation/Pericardial Tamponade	Development of a significant pericardial effusion during or within 30 days of the procedure. A significant pericardial effusion is one that results in:<br>• hemodynamic compromise<br>• requires elective or urgent pericardiocentesis<br>• a 1-cm or more pericardial effusion as documented by echocardiography.
Cerebral infarct or systemic embolism	Rapid onset of a focal or global neurological deficit with at least one of the following:<br>• change in level of consciousness<br>• hemiplegia, hemiparesis, numbness or sensory loss affecting one side of the body<br>• dysphasia or aphasia, hemianopia, amaurosis fugax<br>• other neurological signs or symptoms consistent with stroke<br><br>Duration of a focal or global neurological deficit should be > 24 hours (less if therapeutic intervention(s) were performed (e.g., thrombolytic therapy or intracranial angioplasty)<br>Confirmation of the diagnosis by at least one of the following:<br>• neurology or neurosurgical specialist<br>• neuroimaging procedure (MRI or CT scan or cerebral angiography<br>• lumbar puncture (i.e., spinal fluid analysis diagnostic of intracranial hemorrhage)
Major bleeding requiring transfusion	Significant bleeding that requires and/or is treated with transfusion of blood products (≥ 2 units)

Mitral, Tricuspid or Aortic valve damage resulting in moderate or severe regurgitation	Damage to the leaflets or muscles supporting the valve function
Access site complications requiring medical or surgical intervention	Vascular access complications include development of a hematoma, an AV fistula, or a pseudoaneurysm. A major vascular complication is defined as one that requires pharmacological intervention or surgical repair.
Pericarditis	Inflammatory process that leads to an effusion, causes a hemodynamic compromise, requires a pericardiocentesis, or results in chest pain that persists for 30 days following the ablation procedure.
Heart block requiring a permanent pacemaker	Cardiac conduction block of the AV node requiring the implantation of a permanent pacemaker
Other serious adverse device effects (SADEs), including TIAs, adjudicated by an independent DSMB to be probably or definitely related to the Adagio System	A major complication is a complication that results in permanent injury or death, requires intervention for treatment, or prolongs or requires hospitalization.

VI. Supplemental Figures

Figure 1. Ventricular ULTC lesions in pre-clinical models. Measurements of the lateral (**A**) and cross-sectional (**B**) dimensions of the chronic lesion on gross necropsy. **C**: histological appearance of the chronic ULTC lesion with Mason trichrome stain, showing deep, uniform fibrosis. Courtesy Adagio Medical, Inc. (unpublished)

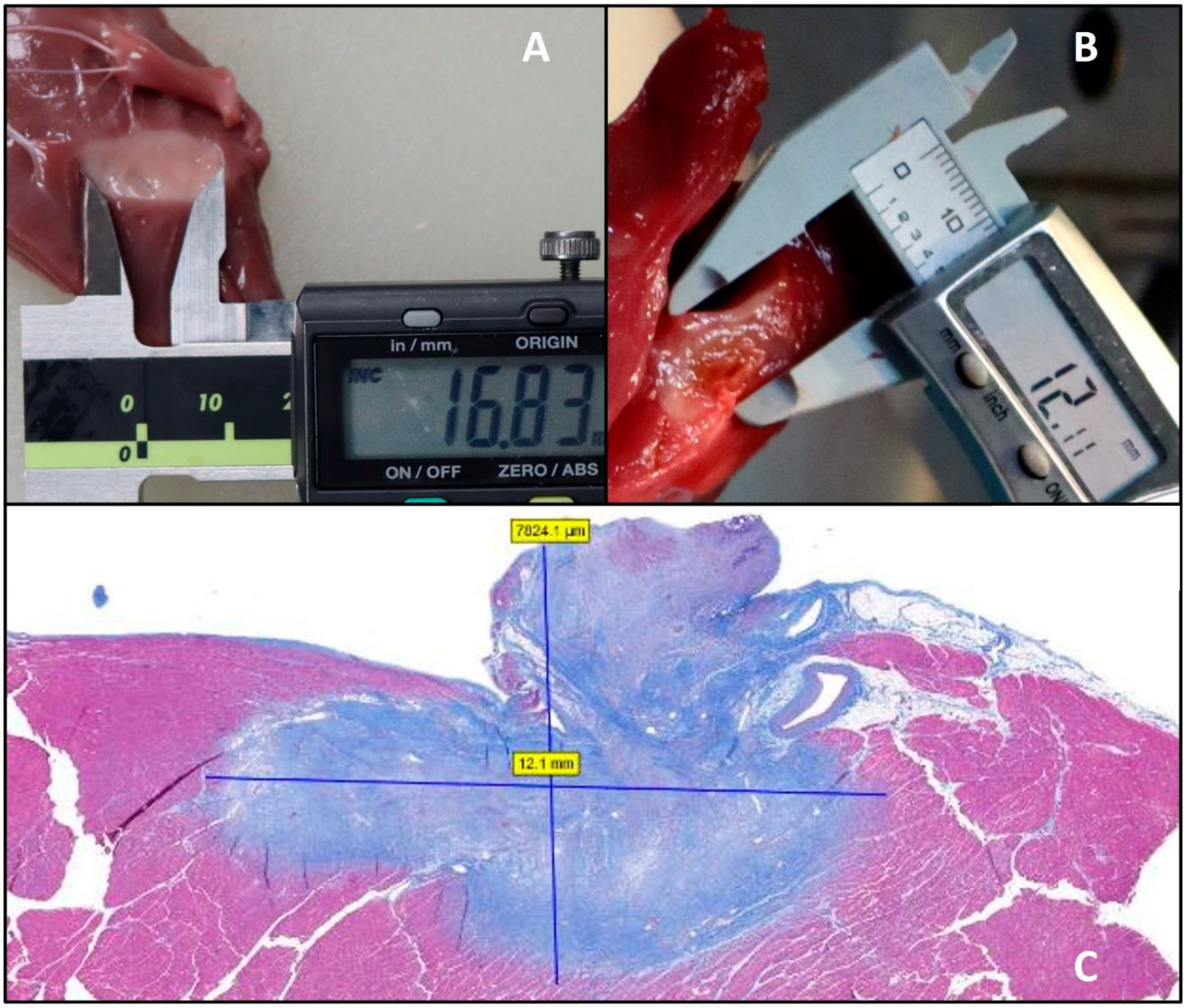

Supplement: euae076_Supplementary_Data [file euae076_supplementary_data.zip › Verma etal Cryocure-VT Europace Supplemental Material .pdf]
